# Advances on intelligent algorithms for scientific computing: an overview

**DOI:** 10.3389/fnbot.2023.1190977

**Published:** 2023-04-21

**Authors:** Cheng Hua, Xinwei Cao, Bolin Liao, Shuai Li

**Affiliations:** ^1^College of Computer Science and Engineering, Jishou University, Jishou, China; ^2^School of Business, Jiangnan University, Wuxi, China; ^3^Faculty of Information Technology and Electrical Engineering, University of Oulu, Oulu, Finland; ^4^VTT Technical Research Centre of Finland, Oulu, Finland

**Keywords:** neural networks, intelligent systems, robotic, dynamic systems, optimization algorithms and strategies

## Abstract

The field of computer science has undergone rapid expansion due to the increasing interest in improving system performance. This has resulted in the emergence of advanced techniques, such as neural networks, intelligent systems, optimization algorithms, and optimization strategies. These innovations have created novel opportunities and challenges in various domains. This paper presents a thorough examination of three intelligent methods: neural networks, intelligent systems, and optimization algorithms and strategies. It discusses the fundamental principles and techniques employed in these fields, as well as the recent advancements and future prospects. Additionally, this paper analyzes the advantages and limitations of these intelligent approaches. Ultimately, it serves as a comprehensive summary and overview of these critical and rapidly evolving fields, offering an informative guide for novices and researchers interested in these areas.

## 1. Introduction

In recent years, the fields of computer science and communication electronics have undergone rapid growth and development, primarily due to the increasing interest in techniques that can enhance the performance of systems. The advancement of technologies such as neural networks, intelligent systems, optimization algorithms, and strategies has resulted in significant progress and created new opportunities and challenges in the areas of artificial intelligence, automation, and data science.

Neural networks, a potent machine learning algorithm, have garnered considerable attention due to their ability to solve intricate problems in diverse fields, such as speech recognition, image processing, and reinforcement learning. Inspired by the human brain's structure, neural networks consist of interconnected layers of nodes or “neurons” that process input data and generate output predictions. The primary advantage of neural networks stems from their self-learning capability, which enables them to assimilate knowledge from vast amounts of data and make accurate predictions without explicit programming. Consequently, they find extensive applications in domains where traditional programming is arduous and cumbersome. Additionally, neural networks can handle non-linear relationships between inputs and outputs, rendering them highly suitable for complex non-linear problems that are challenging to solve with linear models. However, neural networks also possess certain limitations, such as: (1) Black-box nature: Neural networks are often regarded as black-box models due to the challenge in comprehending how they arrive at their prediction outcomes. Consequently, diagnosing and rectifying errors in the model can be difficult; (2) Overfitting: Neural networks are susceptible to overfitting, which implies that they may perform well on the training data but poorly on new and unseen data. This can be mitigated by utilizing regularization techniques, but it continues to pose a challenge. (3) Training complexity: Neural networks are computationally intensive and time-consuming to train, particularly for large and complex datasets. In general, neural networks are potent tools in the realm of machine learning and have demonstrated considerable potential in solving intricate problems (Xiao et al., 2018b; Long et al., 2022; Peng and Liao, 2022; Liao et al., 2023). With sustained research efforts and continued development, they may offer even greater utility across a broad range of applications.

Intelligent systems have evolved into a pervasive and indispensable element of modern society. These systems utilize artificial intelligence and electronic communication technology to provide solutions for diverse applications, ranging from self-driving cars to home automation systems (Khan et al., 2022e). The widespread implementation of intelligent systems can be attributed to the steady advancement of technologies such as design, recognition, detection, prediction, and evaluation. Furthermore, the exceptional performance of intelligent system components, including communication systems and oscillators, assumes a crucial role. Communication systems are indispensable for transmitting data and commands between distinct components of the system (Zhang et al., 2022a), while oscillators provide accurate timing and synchronization to ensure the proper operation of the system (Jin et al., 2017a).

Optimization represents a fundamental challenge in multiple domains, entailing the identification of the optimal solution to a problem that complies with prescribed criteria and constraints. Optimization algorithms and strategies seek to automate this process and attain the optimal solution efficiently. Over time, diverse optimization algorithms have been developed, which can be broadly categorized into classical and metaheuristic approaches. Classical methods rely on mathematical techniques such as linear programming (Hu et al., 2019a), quadratic programming (Xiao, 2016; Xiao et al., 2019c), and dynamic programming (Lv et al., 2018; Liao et al., 2019), while metaheuristic methods are more heuristic and often inspired by natural phenomena (Sun et al., 2016; Khan et al., 2020a; Qu et al., 2020; Zhang et al., 2022b). Optimization methods and strategies play a critical role in the efficacy and competitiveness of various fields (Khan et al., 2021). For instance, optimization technologies can be employed to enhance the performance of machines or systems while reducing costs. Furthermore, optimization methods can have a favorable impact on society by improving the efficiency of public services and infrastructure, and addressing societal challenges such as poverty, inequality, and climate change. Overall, optimization methods and strategies constitute a crucial aspect from all perspectives.

This paper aims to present a comprehensive survey of three areas of research: neural networks, intelligent systems, and optimization algorithms and strategies. The basic principles, techniques, recent advances, and future directions of these intelligent methods will be explored in depth. This paper will provide a detailed examination of the models, algorithms, and applications used in each of these research fields. Furthermore, the advantages and limitations of these technologies will be thoroughly analyzed and discussed to aid readers in understanding and evaluating these intelligent methods. The structure of this paper is presented as follows. In Section 2, we categorize neural network models into real-valued and complex-valued types, and examine the activation function, robustness, and convergence of these models. Moreover, this section illustrates the relevant application domains of neural networks, including linear systems, non-linear systems, and robotic and motion planning. Section 3 discusses the pertinent technologies and components of intelligent systems, comprising system design, recognition, and detection methods, prediction and evaluation methods, and intelligent communication systems and oscillators. In Section 4, we explore bio-inspired optimization algorithms and optimization strategies and systems. Finally, Section 5 provides concluding remarks.

## 2. Neural networks

### 2.1. Background

**Figure 1:**
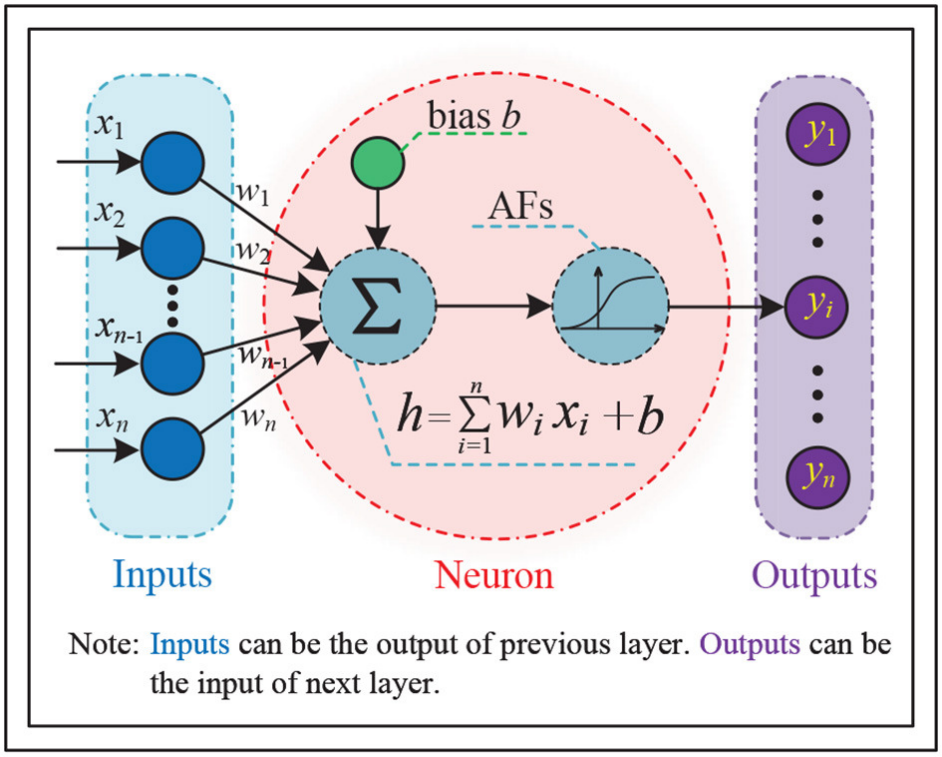
General structure of a single neuron in the most basic type of neural networks, where *x*_*i*_ denotes the *i*th input of the neuron, *w*_*i*_ is the corresponding weight, *y*_*i*_ represents the *i*th output of the neuron, and the activation functions (AFs) can be linear or non-linear.

Neural networks are mathematical models that simulate the processing of complex information by the human brain's nervous system, based on the principles of neural networks in biology. These models abstract the structure of the brain and its response mechanism to external stimuli, and are represented by a large number of interconnected nodes (called neurons) with specific output functions (called activation functions or AFs). Connections between nodes represent weighted values (called weights) for signal transmission, allowing neural networks to simulate human memory. The network's output depends on its structure, connections, weights, and activation functions, which are typically approximations of algorithms, functions of nature, or logical strategies. Figure 1 illustrates the structure of a single neuron in the most basic type of neural network.

The neural network model has gained significant attention across various scientific domains due to its distinctive properties, which are as follows:

- **Self-learning and self-adaptive ability:** The neural network model is capable of adjusting its network structure parameters automatically when exposed to changes in the external environment (such as new training samples), to achieve the desired output corresponding to a specific input. Compared to traditional expert systems with fixed reasoning, neural network models are more adaptable and mimic the thinking style of the human brain.
- **Non-linearity:** Many real-world problems are viewed as non-linear complex systems, while neural networks store information in the number of neurons and connection weights, allowing for various non-linear mappings.
- **Fault-tolerance and robustness:** The distributed nature of information storage in neural network models ensures that local damage to the model moderately weakens the operation of the neural network without producing catastrophic errors. Moreover, neural networks can handle incomplete or noisy data, possess generalization function, and exhibit strong fault tolerance.
- **Computational parallelism and distributed storage:** The structural features of neural networks result in natural parallelism. Each neuron can perform independent operations and processing based on the received information and output the result. Different neurons in the same layer can perform operations simultaneously and then transmit to the next layer for processing. As a result, neural networks can take advantage of parallel computing to increase their operation speed significantly. Neural networks use distributed storage to represent information. By distributing the activation signals on the network neurons in response to the input information, the features are accurately remembered in the connection weights of the network through training and learning, enabling the neural network to make quick judgments when the same patterns are input again.

In the preceding subsection, we have acquired an initial comprehension of the fundamental architecture and characteristics of neural network models. In the following analysis, we will examine the models in greater detail from the standpoint of their various categories, problem-solving approaches, and practical applications.

### 2.2. Real-valued neural network model

Real-valued neural networks are a type of machine learning model that can process continuous data, making them highly versatile and effective in various domains, such as computer vision, natural language processing, and signal processing. For example, in image recognition, real-valued neural networks can take the pixel values of a digital image as input and produce the corresponding label as output. In stock price prediction, these networks can model historical stock data and provide trend predictions for future stock prices. In voice recognition, acoustic signals can be transformed into textual output through the use of real-valued neural networks. The activation function (AF) is a crucial component of the neural network architecture as it enables the transformation of the input into an output. Without an AF, the neural network can only represent linear functions. The addition of a non-linear AF allows the neural network model to achieve non-linear transformations from input to output, thereby enhancing its expressive power.

#### 2.2.1. Neural network model with linear AF

Let us first consider the neural network model with a linear AF. In this case, the gradient, or derivative, of the neural network remains constant for each iteration, making it difficult for the model to capture complex information from the data. However, linear AF is still suitable for simple tasks that require high interpretability. In their study (Ding et al., 2014), the authors proposed a class of static recurrent neural network (SRNN) models with linear activation function and time-varying delays. To assess the stability of the SRNN model, they introduced a new Lyapunov-Krasovskii function and derived improved time delay-dependent stability conditions in the form of linear inequalities. They then provided numerical results that are consistent with the theoretical findings by specifying the SRNN model parameters. In another study (Zhang et al., 2019), the authors extended the original linearly activated fixed-parameter neural network to a linearly activated varying-parameter neural network model, where the parameter is chosen as ζ(*t*) = α+α^*t*^. Subsequently, Xiao et al. proposed an improved varying parameter neural network model (Xiao et al., 2020c). The parameter value of this model is


$$\zeta (t)=\{\begin{array}{ll} \alpha +t^{\alpha }, & \text{if 0}<\alpha \leq \text{1},\\ \alpha^{2}+2t\alpha +\alpha^{t+2}, & \text{if }\alpha >\text{1}, \end{array}$$

which can better meet the needs of the model hardware implementation.

The integration of various neural network approaches has garnered significant interest in addition to the investigation of individual neural network models. A novel strategy combining gradient-based neural networks (GNNs) and zeroing neural networks (ZNNs) was proposed in Dai et al. (2022) to solve dynamic matrix inversion online. The proposed strategy incorporates fuzzy adaptive control, which allows for adaptive adjustment by regulating the fuzzy factors based on real-time residual error values. The authors demonstrate the global convergence and efficacy of this GNN-ZNN model based on fuzzy control through theoretical analysis and numerical experiments. Different papers have employed various neural network models for the same problem, each with their own unique characteristics (Zhang et al., 2019; Xiao et al., 2020c; Dai et al., 2022). Therefore, exploring how to effectively combine the strengths of multiple neural network models in different scenarios is an important area of research. Fuzzy control theory, a mathematical theory dealing with fuzziness, is based on the concept of fuzzy sets and has been widely studied, including applications such as fuzzy inference (Zeng et al., 2022) and fuzzy Petri nets (Zhou et al., 2015, 2018a,b, 2019). These fuzzy control methods offer guidance for extending single neural networks to multi-neural networks.

#### 2.2.2. Neural network model with non-linear AF

Non-linear AFs are a crucial element of neural networks, contributing to their expressive power and learning capability, leading to superior performance in handling complex tasks. Based on convergence properties, non-linear AFs can be categorized into two types: general AFs and finite-time convergent AFs.

(i) **General AFs:** In recent years, several studies have proposed neural network models with non-linear activation functions for solving a variety of problems. For example, in Jian et al. (2020), a class of neural network models was presented for solving the time-varying Sylvester equation, where the authors considered three different types of non-linear activation functions and provided a detailed theoretical derivation to validate the convergence performance of the proposed models. Similarly, Lei et al. proposed an integral structured neural network model with a coalescent activation function optimized for the solution of the time-varying Sylvester equation (Lei et al., 2022). For non-convex and non-linear optimization problems, an adaptive parameter convergence-differential neural network (CDNN) model with non-linear activation functions was proposed in Zhang et al. (2018d), and the authors verified the global convergence and robustness of the model by theoretical analysis and numerical experiments. Non-linear activation functions are also widely used in many fields, such as wheeled mobile robot control (Xiao et al., 2017b), surgical endoscopic robot control (Li et al., 2022b), and distributed collaborative networks (Zhang et al., 2018a).

(ii) **Finite-time convergent AFs:** Contrary to the general non-linear activation functions with infinite time convergence, the activation functions with finite time convergence facilitate fast convergence of neural network models, with a time upper bound. In Xiao et al. (2018a), the authors proposed a neural network model for online solution of Lyapunov equations in non-linear systems. The model's fast convergence was achieved by incorporating non-linear activation functions, and an upper bound on the model's time convergence was established via theoretical analysis as


$$\begin{array}{l} {\text{Time}}_{\text{up}}<\frac{\alpha_{1}+\beta_{1}}{\alpha_{1}\beta_{1}(1-\zeta )}\text{max}\left\{|r^{-}(0){|}^{\left(1-\zeta \right)},|r^{+}(0){|}^{\left(1-\zeta \right)}\right\}, \end{array}$$

where α_1_ and β_1_ are scale factors, ζ ∈ (0, 1), *r*^+^(0) = max{*R*(0)}, and *r*^−^(0) = min{*R*(0)} with *R*(0) denotes the initial value of the error function *R*(*t*). Finally, the stability and finite-time properties of the model were confirmed in an application involving the control of a six-link robotic arm. In a similar vein, Xiao et al. developed an accelerated convergence recurrent neural network (RNN) model (Xiao, 2017a, 2019) for time-varying matrix square root finding (Zhang et al., 2015), and provided a time upper bound for the convergence of the model, which is expressed as


$$\begin{array}{l} {\text{Time}}_{\text{up}}=\frac{\alpha_{2}}{\beta_{2}\left(\alpha_{2}-\gamma \right)}\ln\frac{\beta_{2}A{\left(0\right)}^{\left(\alpha_{2}-\gamma \right)/\alpha_{2}}+\lambda }{\lambda }, \end{array}$$

where β_2_ and λ are scale factors, α_2_ > γ and all are odd integers, and *A*(0) is a random initial value of the error matrix. For dynamic non-linear optimization problems (Liao et al., 2015; Xiao and Lu, 2019; Lu et al., 2020), the authors proposed a sign-bi-power AF and use it for dynamic neural network model design, and express the upper bound of the model convergence time mathematically as


$${\text{Time}}_{\text{up}}<\text{max}\left\{\frac{|k_{0}^{+}{|}^{\left(1-\alpha_{3}\right)}}{\beta_{3}(1-\alpha_{3})},\frac{|k_{0}^{-}{|}^{\left(1-\alpha_{3}\right)}}{\beta_{3}(1-\alpha_{3})}\right\}$$

where α_3_ is the scale factor, 1 < β_3_ < 1, $k_{0}^{+}$ and $k_{0}^{-}$ represent the maximum and minimum initial values of the error vector ***k***, respectively. In order to account for the effects of rounding errors and external noise disturbances in practical problem solutions, Xiao and colleagues proposed a neural network model in Xiao et al. (2019d) with the capability to suppress noise and achieve predefined time convergence. The authors provided detailed theoretical proof of the robustness and finite-time convergence of the model. They also verified through numerical experiments that the model can still achieve finite-time convergence in the presence of external noise. In Liao et al. (2022a), a predefined time-convergent neural network model with harmonic-like noise suppression was designed for adaptively solving time-varying problems by leveraging the properties of harmonic signals. The burgeoning demand for real-time performance has become a critical requirement for many scientific, industrial, and commercial applications, such as computational biology, weather forecasting, autonomous vehicles, and financial analytics. This requirement is largely driven by the rapid progress in computer technology, including advances in hardware and software, which have enabled the processing of vast quantities of data in real-time (Tan and Dai, 2017; Dai et al., 2018; Tan, 2021; Li et al., 2022a). Real-time performance is essential for many time-sensitive applications, where delays or inaccuracies in processing can have severe consequences, such as in real-time monitoring of critical physiological signals or detecting anomalies in sensor data. Furthermore, real-time performance enables immediate feedback and adaptive decision-making, leading to increased efficiency and performance. In Zhang et al. (2022c), the authors proposed a unified GNN model for handling both static matrix inversion and time-varying matrix inversion with finite-time convergence and a simpler structure. As the authors conclude, compared with the existing GNN model and ZNN model dedicated to time-varying matrix inversion, the proposed unified GNN model has advantages in convergence speed and robustness to noise. At the same time, the authors further extend this GNN model for finding the dynamic Moore-Penrose inverses in real-time (Zhang et al., 2022d), and the paper concludes that this method does not require the time derivatives of the relevant dynamic matrices and has finite time convergence. In short, high-precision and low-complexity real-time solutions are a highly active area of research, with numerous open problems and opportunities for innovation in both fundamental algorithms and system-level optimizations.

To facilitate the reader's understanding, we present a list of the linear and non-linear activation functions discussed in Section 2 and provide a detailed description of each function in Table 1. (1) Linear activation function (LAF):


(1) $$\begin{array}{l} A\left(x\right)=x. \end{array}$$

(2) Power activation function (PAF):


(2) $$\begin{array}{l} A\left(x\right)=x^{\mu }\;\text{with }\mu >3\;\text{indicating an odd integer}. \end{array}$$

(3) Bipolar sigmoid activation function (BPAF):


(3) $$\begin{array}{l} A\left(x\right)=\left(1-\text{exp}\left(-\mu x\right)\right)/\left(1+\text{exp}\left(-\mu x\right)\right)\;\text{with }\mu >1. \end{array}$$

(4) Power-sigmoid activation function (PSAF):


(4) $$A(x)=\{\begin{array}{ll} x^{\mu }, & \text{if |x|}\geq \text{1},\\ \frac{1-\text{exp}(-\mu x)}{1+\text{exp}(-\mu x)}\cdot \frac{1+\text{exp}(-\mu )}{1-\text{exp}(-\mu )}, & \text{otherwise}. \end{array}$$

(5) Hyperbolic sine activation function (HSAF):


(5) $$\begin{array}{l} A\left(x\right)=\left(\text{exp}\left(\mu x\right)-\text{exp}\left(-\mu x\right)\right)/2\;\text{with }\mu >1. \end{array}$$

(6) Sign-bi-power activation function (SBPAF) :


(6) $$\begin{array}{l} A\left(x\right)=\left(|x{|}^{\mu }+|x{|}^{1/\mu }\right)\text{sgn}\left(x\right)/2\;\text{with }0<\mu <1, \end{array}$$

thereinto,


$$\text{sgn}(x)=\{\begin{array}{ll} 1, & \text{if }x>\text{0},\\ 0, & \text{if }x\text{ = 0},\\ -1, & \text{if }x<\text{0}. \end{array}$$

7) Tunable sign-bi-power activation function (TSBPAF):


(7) $$\begin{array}{l} A\left(x\right)=\frac{1}{2}\rho_{1}|x{|}^{\mu }\text{sgn}\left(x\right)+\frac{1}{2}\rho_{2}x+\frac{1}{2}\rho_{3}|x{|}^{1/\mu }\text{sgn}\left(x\right), \end{array}$$

where μ ∈ (0, 1), ρ_1_, ρ_2_, and ρ_3_ are greater than 1.

**Table 1:** Details of various linear and non-linear activation functions.

**AFs**	**Type**	**References**
LAF (1)	Linear	(Ding et al., 2014; Zhang et al., 2019; Jian et al., 2020; Xiao et al., 2020c; Dai et al., 2022)
PAF (2)	Non-linear	(Jian et al., 2020)
BPAF (3)	Non-linear	(Zhang et al., 2018a; Lei et al., 2022)
PSAF (4)	Non-linear	(Zhang et al., 2018d)
HSAF (5)	Non-linear	(Xiao et al., 2017b; Li et al., 2022b)
SBPAF (6)	Non-linear & Finite-time convergence	(Xiao, 2017a, 2019; Xiao et al., 2018a, 2019d)
TSBPAF (7)	Non-linear & Finite-time convergence	(Liao et al., 2022a)

### 2.3. Complex-valued neural network model

In recent years, neural network-based machine learning techniques have found broad application in practical settings. Notably, the majority of current neural network models are designed for real-valued inputs, outputs, and weights. However, this raises the question of the existence and purpose of complex-valued neural network models. What are complex-valued neural network models, and why are they necessary? Complex-valued neural network models utilize complex numbers as inputs, outputs, and weights and are inspired by the natural properties of complex numbers and the existence of complex-valued neurons in biology. They are employed in specific application scenarios where the input and output data can be represented in complex form, and therefore, complex-valued neural networks can better describe and process these data. Compared to real-valued neural networks, complex-valued neural networks offer several advantages:

- They can better represent complex-valued data in the real world, such as sound waves and electromagnetic waves.
- They can achieve better results with a smaller network size due to the effectiveness of complex-valued weights in expressing correlations and symmetries in the data.
- They can better handle asymmetrical data by allowing for expression rotation and scaling, which can map asymmetric data into a more symmetric space.
- They can better handle phase information, which is important for complex-valued data, as traditional real-valued neural network models struggle to handle the phase information effectively.

Complex-valued neural networks have been extensively employed in image recognition, speech recognition, and natural language processing, and are currently under thorough investigation. In the following sections, we will delve into the complex-valued neural network model and scrutinize it through the lenses of noise-tolerance and finite-time convergence.

#### 2.3.1. Noise-tolerance

The precision and robustness of neural network models can be adversely affected by computational rounding errors and external noise perturbations. Therefore, it is crucial for these models to possess the dual capability of solving problems and suppressing noise simultaneously.

In Xiao and Lu (2017), a complex-valued gradient neural network model was proposed for solving complex-valued linear matrix equations. This model has a simpler theoretical analysis and lower computational complexity compared to the widely used real-valued gradient-based neural network model. In Lei et al. (2020), the authors proposed a neural network model for computing the inverse of complex-valued time-varying matrices. The model's convergence in solving time-varying problems and its robustness against external noise disturbances were analyzed and validated. The effect of design parameters on the speed of model solving was also elucidated based on experimental results. Moreover, a complex-valued noise-resistant neural network model based on an integral-type design formulation was presented in Xiao et al. (2019f) for the same problem. The convergence and robustness of the model were verified through detailed analysis and proofs. The experiments considered various noise types, including constant noise, linear noise, bounded linear noise, harmonic noise, and exponential-type noise. The model proposed in this work has a better noise suppression effect compared to the traditional gradient-based neural network model. To further improve the noise tolerance of the neural network model, a complex-valued noise-tolerant neural network model with a double-integral structure was proposed in Liao et al. (2022b), which was capable of simultaneously solving the problem and suppressing the noise. The authors verified the robustness of the model under constant noise, linear polynomial noise, and quadratic polynomial noise via numerous theoretical analyses. According to the numerical experimental results, this model can achieve the effective suppression of constant noise, linear polynomial noise, and quadratic polynomial noise. In Ding et al. (2019b), Ding et al. proposed an improved complex-valued recurrent neural network (ICVRNN) model for solving the complex-valued time-varying Sylvester equation. This work gives a large number of theoretical proofs and experimental cases to analyze the effectiveness, convergence, and stability of the ICVRNN model. Additionally, the authors further extend this ICVRNN model to the solution of complex-valued linear equations (CVLEs) (Ding et al., 2018). As the authors conclude, the ICVRNN model has better performance for solving CVLEs compared to traditional neural network models. In addition, noise-tolerant complex-valued neural network models are widely used for solving many problems, such as matrix pseudo-inverse solving (Lei et al., 2019), robotics (Liao et al., 2022d), and non-linear optimization (Xiao et al., 2019a), etc.

#### 2.3.2. Finite-time convergence

Finite-time convergence is a crucial characteristic of neural network models as it allows for achieving the desired level of performance in a shorter amount of time. Specifically, if a neural network model can attain convergence within a finite time, the parameter selection and tuning process can be expedited to obtain the desired results more quickly. The non-linear activation function used in complex-valued neural network models plays a pivotal role in achieving finite-time convergence. This function is based on the non-linear activation function in the real domain but generalized to the complex domain. Unlike its counterpart in the real domain, the complex-valued non-linear activation function operates on complex inputs and outputs, which enables better handling of the non-linear characteristics of complex-valued data.

In Li and Li (2013), Li et al. proposed two ways to generalize the AF from the real domain to the complex domain, as follows.

i) **Complex-valued AF Type I:**


$$\begin{array}{l} F\left(a+ib\right)=A\left(a\right)+iA\left(b\right), \end{array}$$

where F(·) is a complex-valued AF defined in an element-wise manner, and *a* and *b* denote the real and imaginary parts of the complex number *a* + *bi*, respectively.

ii) **Complex-valued AF Type II:**


$$\begin{array}{l} F\left(a+ib\right)=A\left(\text{Υ}\right)◇\text{exp}\left(i\text{Θ}\right), \end{array}$$

where the symbol ◇ denotes the multiplication of the corresponding subelements of two vectors or matrices (i.e., ***c*** ◇ ***d*** = [*c*_*j*_*d*_*j*_] for real vectors ***c*** = [*c*_*j*_] and ***d*** = [*d*_*j*_]), and Υ ∈ ℝ and Θ ∈ (−π, π] represent the modulus and argument of the complex number *a* + *bi*, respectively.

In Xiao et al. (2020b), the authors proposed two non-linear equivalent models for solving complex-valued problems. One model focused on the real and imaginary parts of the complex numbers, while the other was from the perspective of the modulus of the complex numbers. The authors introduced a non-linear activation function to ensure fast convergence and applied these models to solve the complex-valued Sylvester equation. Both models performed well, as reported by the authors. In Xiao et al. (2022b), the authors designed an arctan-type variable-parameter complex-valued neural network model with finite-time convergence. This model takes into account the reality that the convergence factor is time-varying in the actual hardware environment. During the solution process, the model can adjust its convergence scale parameters (CSPs). When the model achieves convergence, the CSPs converge to a constant greater than zero. The CSPs and finite-time upper bounds of this model are supported by theoretical analysis, as the authors conclude. The excellent performance of this model has been demonstrated in numerical experiments. Furthermore, the authors extended this variable-parameter neural network model to solve time-varying complex-valued matrix equations (Ding et al., 2018; Xiao et al., 2021b).

In Zhou et al. (2022), the authors aimed to improve the robustness and solution speed of complex-valued noise-resistant neural network models for practical problem-solving, while meeting the dual requirements of noise tolerance and real-time performance. To this end, the authors introduced non-linear activation to the model. In this work, the authors employed this improved model to solve the problem of trajectory tracking for manipulators, and the results demonstrate that this model can effectively suppress noise while meeting real-time requirements of the task. In another work (Xiao et al., 2021a), the authors utilized a complex representation to convert the quaternion-valued matrix into the corresponding time-varying complex-valued matrix (TVCVM), and then proposed a complex-valued neural network model to solve this TVCVM. The authors introduced a versatile non-linear-sign activation function to achieve the predefined time convergence of the model. According to the authors' summarized results, theoretical analysis provided an upper bound for the convergence time of this model. Finally, the authors applied this model to a mobile manipulator and demonstrated its good performance.

### 2.4. Neural networks for linear system solving

A linear system is characterized by the linear property, which states that the system response is homogeneous and additive, such that the output signal changes in proportion to the input signal of the system. Solving linear systems with neural networks is of significance as it enables fast processing via learning and optimization, particularly for problems that are difficult or computationally complex to solve by traditional methods. Compared to traditional solution methods, using neural networks to solve linear systems has the following advantages.

- **Strong solving ability:** It can handle large-scale, high-dimensional linear systems, where traditional methods may be computationally overloaded or numerically unstable.
- **Good adaptability:** It can adaptively learn the mapping relationship between input and output, this allows neural networks for more complex linear system solving.
- **High accuracy in solving:** It can improve the accuracy of the model by increasing the number of layers and neurons of the neural network, this makes the neural network applicable to the solution of linear systems with high accuracy requirements.

#### 2.4.1. Linear equation

In many real-time applications, including control and signal processing, precise analysis and control of linear systems are crucial. To this end, various neural network models have been proposed for the online solution of time-varying linear systems. For instance, in Lu et al. (2019), the authors introduced a novel recurrent neural network (RNN) model for solving time-varying underdetermined linear systems while satisfying the constraints of state variables and residual errors. This work presented extensive theoretical analyses and numerical cases to demonstrate the effectiveness and validity of the proposed RNN model, which was further applied to control the PUMA560 robot under physical constraints. In Xiao et al. (2019b), the authors developed a neural network model for time-varying linear matrix equations and provided a theoretical analysis of the upper bound on the time convergence of the model. The study concluded that this model demonstrated exceptional performance in solving time-varying linear equations. Additionally, in Zhang et al. (2018b), the authors proposed a varying-gain RNN model for solving the linear system *H*(*t*)*J*(*t*)*K*(*t*) = *L*(*t*), with the design parameters of the model being characterized by time-varying properties. The finite-time convergence of this model was also verified by theoretical analysis. In Xiao et al. (2019e), two non-linear neural network models were investigated for solving the dynamic Lyapunov equation *H*^T^(*t*)*J*(*t*) + *J*(*t*)*H*(*t*) = −*K*(*t*), and the study noted that the solution outcomes of these models were independent of the choice of initial values. Similarly, in Xiang et al. (2018a), the authors proposed a discrete Z-type neural network (DZTNN) model for the same dynamic Lyapunov equation, which exhibited inherent noise tolerance and exact solution attainment under various types of noise. Additionally, various neural network models (Xiao, 2017b; Jin et al., 2019; Xiao and He, 2021; Lei et al., 2022; Han et al., 2023) have been put forward for solving the time-varying Sylvester equations *H*(*t*)*J*(*t*)−*J*(*t*)*H*(*t*) = −*K*(*t*).

#### 2.4.2. System of linear equations

The system of linear equations is a fundamental mathematical concept used in various fields as a powerful tool to solve practical problems due to its linearity, simultaneousness, infinite solutions, and suitability for multiple methods. In Xiao et al. (2022a), the authors proposed a neural network model with adjustable parameters and demonstrated its fast convergence speed, low upper limit of convergence time, and short parameter adjustment time. The study also applied the model to achieve synchronous control of chaotic systems and validated its effectiveness. The authors concluded that this model performed excellently. In Xiao et al. (2017a), a gradient-based dynamic model was proposed for the simultaneous solution of systems of linear equations. The authors demonstrated that the model had a zero error bound at convergence and provided an upper bound on the convergence time. Additionally, this class of dynamic models was extended to the online solution of complex-valued systems of linear equations (Xiao, 2015; Xiao et al., 2021b). To meet the requirements of high real-time and strong robustness in solving linear systems of equations in engineering practice, in Xiao et al. (2020a), the authors developed a dynamic control model with noise robustness for online solution of systems of linear equations. The paper designed a non-linear activation function with noise tolerance and added it to the dynamic control model. The authors theoretically analyzed the noise immunity, convergence, and robustness of the model. Furthermore, the authors applied the dynamic control model to the motion tracking of the robot, and the results demonstrated good performance in the elliptical path tracking control of the robot. In Katsikis et al. (2023), the authors proposed a dynamic neural network model, based on neutrosophic numbers and a neutrosophic logic engine, which exhibits superior performance compared to the traditional ZNN design. The primary objective of this model is to estimate the matrix pseudo-inverse and minimum-norm least-squares solutions of time-varying linear systems. The observed enhancement in efficiency and accuracy of the proposed model over existing techniques is attributed to the advantages of neutrosophic logic over fuzzy and intuitionistic fuzzy logic. The authors utilized neutrosphication, de-fuzzification, and de-neutrosophication instead of the conventional fuzzification and de-fuzzification methods. The efficacy of the proposed model was assessed through simulation examples and engineering applications in the domains of localization problems and electrical networks.

### 2.5. Neural networks for non-linear system solving

Non-linear systems present a significant challenge for modeling, analysis, and control because their output cannot be described simply by a linear relationship with the input, and their dynamics may exhibit complex behaviors such as chaos or periodicity. The study of non-linear systems is critical to many fields, including control engineering (Xiao et al., 2017b; Zhou et al., 2022), signal processing (Jin, 2014; Luo and Xie, 2017), dynamics analysis (Tan and Dai, 2016; Tan et al., 2017, 2019a; Lu et al., 2020), and communication systems (Jin and Yu, 2012; Jin and Fu, 2013; Jin et al., 2015b; Zhao et al., 2020; Xiang et al., 2022), owing to the following properties.

- **Abundant kinetic behavior:** Unlike linear systems, the kinetic behavior of non-linear systems can be very abundant and diverse. For example, they can generate chaotic phenomena, periodic oscillations, and stable immobile points, etc.
- **Better modeling of complex phenomena in the real world:** Many natural and social phenomena are non-linear, such as ecosystems, economies, and neural systems. Non-linear systems can simulate these phenomena and provide relevant behavioral information.
- **Available for control and optimization:** Non-linear control theory is an important tool for applying non-linear systems to control and optimize problems. For example, in robotics and industrial control, non-linear control enables highly accurate and efficient solving of tasks.

In particular, non-linear systems can exhibit sensitivity to initial conditions, bifurcations, and singularities, making them a rich area of investigation for researchers. Furthermore, non-linear systems are capable of representing a wide range of phenomena, including self-organization, emergence, and adaptation, which are not captured by linear models. Thus, developing effective methods for modeling, analysis, and control of non-linear systems remains an important area of research in many disciplines.

Neural network methods are a powerful tool for real-time parallel processing that can be utilized to solve challenging non-linear systems, particularly for situations in which an analytical solution is elusive. These methods have found application in various domains, including non-linear control problems (Xiao et al., 2019g; Li et al., 2020c; Jia et al., 2021), non-linear differential equations (Zhang et al., 2017, 2018d; Liao et al., 2021), and non-linear optimization problems (Liu et al., 2016; Lan et al., 2017; Xiao et al., 2019a; Zhang et al., 2020).

#### 2.5.1. System of non-linear equations

Non-linear systems frequently appear in real-world applications, and the online solution of systems of non-linear equations has been a subject of extensive research. One popular approach for solving such systems is through the use of neural network methods, which can be particularly useful when the analytical solution is difficult to obtain. In Xiao et al. (2019g), the authors proposed a class of recurrent neural network (RNN) models with finite-time convergence for solving systems of non-linear equations. The effectiveness of this RNN model was demonstrated through numerical simulations, and the model was extended to solve more complex non-linear systems, such as the motion tracking control of robotic manipulators. The authors concluded that this RNN model is highly feasible and applicable. Additionally, the authors constructed a discrete noise-resistant recurrent neural network (DNTRNN) model (Li et al., 2020c) based on the five-step finite difference method for the solution of non-linear systems of equations, and demonstrated the effectiveness of the DNTRNN model. In Liu et al. (2016), the authors proposed an RNN model for time-varying non-linear optimization, providing both continuous and discrete forms of the model. The paper concludes that both types of RNN models have superior noise immunity and convergence performance. In Zhang et al. (2018d), the authors designed and proposed a differential neural network with varying parameters and non-linear activation for solving non-convex optimization and non-linear problems online. The global convergence of this neural network model was proven through theoretical analysis, and the authors concluded that this neural network model performs well for solving non-convex and non-linear optimization problems in various numerical experiments.

#### 2.5.2. Quadratic programming (QP)

The quadratic programming method is widely used in practice and is a powerful tool for solving practical problems, which has the following merits.

- **Can describe complex problems:** QP can describe numerous complex optimization problems, such as optimization problems with non-convex functions.
- **Available for constraint handling:** QP can handle optimization problems with constraints, such as inequality constraints, equation constraints, etc. This allows for a broader application of quadratic planning.
- **Extensive solving methods:** The solution methods of QP have been relatively mature, such as the gradient descent method, conjugate gradient method, and neural network method. These methods can be used in practice and can handle large-scale problems.
- **Global optimality:** QP guarantees global optimality for convex quadratic problems, which means that the solution found is guaranteed to be the best possible solution.

Neural network methods offer certain advantages in solving QP problems and are capable of solving large-scale QP problems. Additionally, they avoid the need for mathematical modeling and solving of problems in traditional algorithms. In Liao et al. (2021), the authors introduced neuro-dynamic methods for QP solving and pointed out the limitations of traditional neuro-dynamic methods in the presence of noise. Consequently, they proposed a predetermined time convergence neuro-dynamic method with inherent noise suppression and concluded that this method can achieve a fast and accurate solution to time-varying QP problems in noisy environments. In Zhang et al. (2020), the authors studied a power-type RNN (PT-RNN) model with varying parameters for time-varying QP and quadratic minimization (QM) solving under external perturbations. In this work, the authors provided a detailed design process of this PT-RNN model and analyzed the robustness and convergence of the model theoretically. Lastly, the authors used this model for venture investment and robot tracking. As the authors concluded, this PT-RNN model has great robustness and wide applicability. In Jia et al. (2021), the authors proposed a neural network approach based on an adaptive fuzzy control strategy for time-dependent QP solving. As summarized in the paper, this neural network method can automatically adjust the convergence parameters according to the residual error, which has better results compared with the traditional fixed-parameter neural network method. Similar to QP, non-linear programming (NLP) has also received much attention and is a powerful way to describe complex problems. In Katsikis and Mourtas (2021), the authors aimed to minimize portfolio insurance (PI) costs and presented a multi-period minimum-cost PI (MPMCPI) problem, which incorporates transaction costs, as a more practical version of the classical minimum-cost PI problem. The MPMCPI problem was formulated as a NLP problem, and the authors proposed an approach using intelligent algorithms to solve it. The efficacy of the proposed approach was evaluated using real-world data and compared with other meta-heuristic and commercial methods. The study results contribute to the optimization of portfolio insurance costs using intelligent algorithms and provide insights into the comparative performance of different approaches. Table 2 provides a summary of the works on neural network models for solving linear and non-linear systems.

**Table 2:** Comparison of the properties of neural network models in solving various types of problems.

**Problems**		**Properties of NNs**	**References**
Linear system	Linear equation	Finite-time convergence	(Xiang et al., 2018a; Zhang et al., 2018b; Lu et al., 2019; Xiao et al., 2019b,e; Xiao and He, 2021)
		Noise suppression	(Xiao, 2017b; Xiang et al., 2018a; Jin et al., 2019; Xiao et al., 2019b,e)
	System of linear equations	Finite-time convergent	(Xiao, 2015; Xiao et al., 2017a, 2022a)
		Noise suppression	(Xiao et al., 2020a)
Non-linear system	System of non-linear equations	Finite-time convergent	(Zhang et al., 2018d; Xiao et al., 2019g; Li et al., 2020c)
		Noise suppression	(Liu et al., 2016; Li et al., 2020c)
	Quadratic programming	Finite-time convergent	(Jia et al., 2021; Liao et al., 2021)
		Noise suppression	(Zhang et al., 2020; Liao et al., 2021)

NNs in this table indicate neural networks.

### 2.6. Related applications

Neural networks are widely applied in various fields owing to their parallel computing capability, adaptive learning, and non-linearity. In this subsection, we provide a concise overview of the research on neural networks for redundant robot manipulators. A redundant robot manipulator is a robotic arm that has more degrees of freedom than required. The additional degrees of freedom are known as redundant degrees of freedom. Due to these redundant degrees of freedom, the robotic arm can be more flexibly adapted to different tasks and environments, as well as avoid obstacles or enhance motion performance by adjusting its posture. As a potent tool for real-time parallel processing, neural network models can be used for precise and flexible control of redundant robot manipulators (Xiao and Zhang, 2014; Zhang et al., 2014, 2018c; Liao and Liu, 2015; Jin et al., 2017b; Guo et al., 2018; Tan et al., 2019b; Xiao et al., 2019g; Li et al., 2020d, 2022b; Tang et al., 2022; Zhou et al., 2022). More specifically, neural networks can be used in two ways.

- **Inverse kinematic solving:** The redundant robot manipulator has additional degrees of freedom, and it can move the target position in multiple ways, thus the inverse kinematics needs to be solved to determine the best solution for the motion. Traditional inverse kinematics methods are susceptible to locally optimal solutions, while neural networks can obtain more accurate inverse kinematics solutions by autonomously adjusting the network structure and parameters.
- **Motion planning:** Redundant robot manipulators can use multiple postures to perform the same task, so the optimal sequence of postures needs to be determined for the optimal motion path. Adopting a neural network to solve the optimal posture sequence of the robot manipulator can achieve higher movement efficiency (Khan et al., 2022b).

#### 2.6.1. Inverse kinematic solving

**Figure 2:**
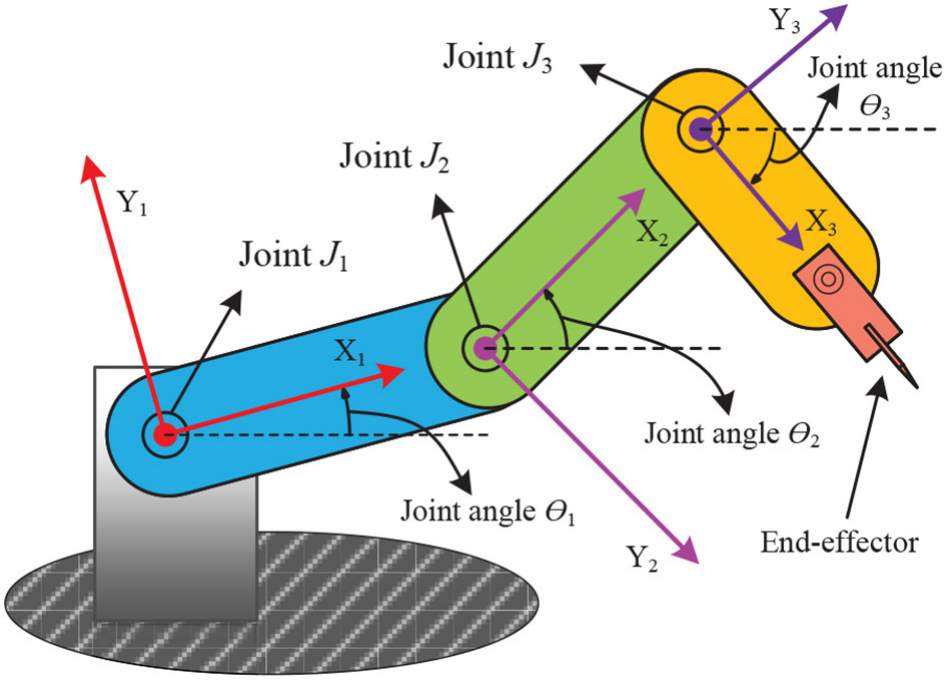
Schematic structure of a three-degree-of-freedom planar robot manipulator.

In Xiao and Zhang (2014), a dynamic neural network model is proposed for solving the inverse kinematics of mobile robot manipulators. The authors provided a theoretical analysis demonstrating the global convergence of the model to the inverse kinematic solution of the mobile robot manipulator, which is also supported by numerical experiments. The paper concludes that this dynamic model outperforms traditional gradient-based neural network models for the inverse kinematic solution of mobile robot manipulators. Liao et al. propose a bi-criteria pseudo-inverse minimization strategy for the redundancy problem of robot manipulators at the joint acceleration level (Liao and Liu, 2015), which can avoid high joint speeds of the manipulator. This method has been validated on a 4-degree-of-freedom robot manipulator and is found to perform well in solving the redundancy problem of robotic manipulators. Tang et al. used an enhanced planning scheme for redundant robot manipulator control (Tang et al., 2022), and a tuning strategy based on this scheme is found to achieve good results in the limit case. Zhang et al. propose a differential scheme with varying parameters for the joint-angle drift (J-AD) problem of redundant robot manipulators (Zhang et al., 2018c). The J-AD problem is formulated as a standard QP problem to be solved, and the authors validate this scheme through computer simulations and physical experiments, concluding that it performs well for solving the J-AD problem of redundant robot manipulators. Figure 2 depicts the schematic structure of a three-degree-of-freedom robot manipulator. In Zhang (2022), the authors discussed the problem of redundancy of manipulators in intelligent systems and designed a dynamic neural network with triple projections, called a tri-projection neural network (TPNN), which is developed for quadratic programs with a constraint on the state evolution of the neuron states. This paper concludes that the TPNN has advantages in fully employing the acceleration capability of the manipulator.

#### 2.6.2. Motion planning

**Figure 3:**
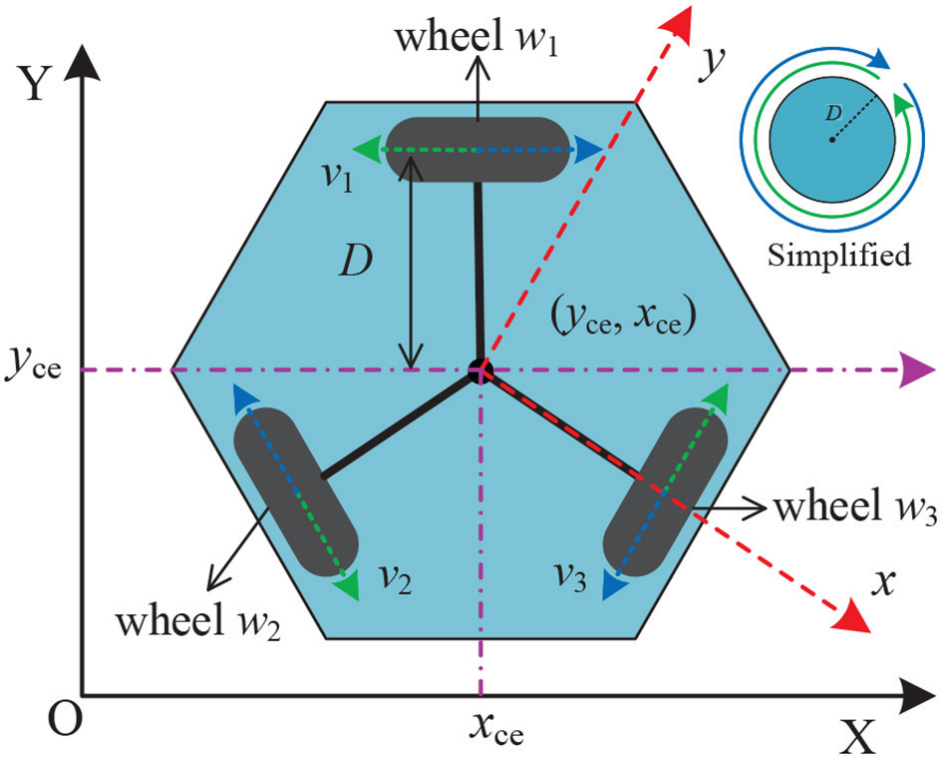
Geometric and kinematic model of an omnidirectional mobile wheeled robot, where (*x*_ce_, *y*_ce_) denotes the geometric center of the wheeled robot.

In Guo et al. (2018), a bi-criteria minimization scheme was proposed for motion planning of redundant robot manipulators, which incorporates joint velocity, joint acceleration, and joint angular constraints into the scheme. The authors design this scheme based on the infinity norm acceleration minimization and minimum weighted velocity criterion. The authors evaluated the scheme through experimental simulations and physical validation, concluding that it is both excellent and physically realizable for redundant robot motion planning. In Jin et al. (2017b), the authors solved the distributed cooperative motion of redundant robot manipulators by reformulating it as a QP problem and designing a neural network model with noise tolerance for this QP problem. The authors validate this neural network model for the problem of the distributed cooperative motion of redundant robotic manipulators in noise-free and noise-containing environments, demonstrating its effectiveness on the PUMA560 redundant robot. Similarly, Li et al. investigated a neural network scheme with noise suppression and use it for redundant robot repetitive motion planning (Li et al., 2020d). The authors verified the effectiveness of this scheme on a four-link and a PA10 robot manipulator, concluding that its performance was superior to conventional motion planning schemes. In Zhang et al. (2014), a QP-based feedback control and motion planning scheme was designed and used for feedback control and motion planning of a mobile robot manipulator. The effectiveness of this scheme has been verified by dynamics analysis, and the authors conclude that it is reliable and superior for feedback control and motion planning of mobile robot manipulators. Figure 3 provides the geometric and kinematic model of an omnidirectional mobile wheeled robot.

### 2.7. Development directions and challenges

In recent years, neural networks have become a dominant technology in machine learning and artificial intelligence. They have achieved state-of-the-art results in various fields, such as image recognition, natural language processing, and game playing. However, neural networks still face several challenges, such as overfitting, data efficiency, and hardware constraints:. In this section, we will discuss the current state and future development directions of neural networks, as well as the challenges that may be faced in the future.

#### 2.7.1. Development directions

Neural networks are expected to evolve in several directions in the future. There are some of the most promising directions:

- **Explainability:** One of the main challenges of neural networks is their lack of interpretability. It is often difficult to understand why a neural network makes a particular decision. Explainable AI (EAI) aims to address this issue by providing human-understandable explanations of the decisions made by neural networks. EAI is expected to become an essential aspect of AI in the future, especially in fields such as healthcare, finance, and autonomous systems.
- **Federated learning:** Federated learning is a distributed machine learning technique that allows multiple parties to collaboratively train a model without sharing their data. It is expected to become increasingly popular in the future due to its privacy-preserving nature. Federated learning can be used in various scenarios, such as personalized recommendation, fraud detection, and predictive maintenance.
- **Quantum neural networks:** Quantum neural networks (QNNs) are a type of neural network that utilizes quantum computation to process information. QNNs have the potential to outperform classical neural networks in various tasks, such as optimization, simulation, and cryptography. QNNs are expected to become increasingly important as quantum computing technology advances.

#### 2.7.2. Challenges

Despite the many advancements in neural networks, they still face several challenges that need to be addressed in the future. There are some of the main challenges:

- **Overfitting:** Overfitting occurs when a neural network learns the noise in the training data instead of the underlying pattern. This can lead to poor generalization performance on new data.
- **Data efficiency:** Neural networks typically require a large amount of labeled data to achieve good performance. This can be a major bottleneck in real-world applications, especially in domains where data is scarce or expensive to obtain. One potential solution to this challenge is the development of transfer learning techniques that allow pre-trained models to be fine-tuned on smaller datasets.
- **Hardware constraints:** Neural networks require large amounts of computation and memory resources, which can be challenging to deploy on resource-constrained devices such as mobile phones and IoT devices. One potential solution is the development of hardware optimized for neural network computations, such as specialized processors and accelerators.

## 3. Intelligent systems

An intelligent system is an automated system that leverages computer and artificial intelligence technology to enable intelligent decision-making, control, and management. It facilitates automatic control and optimization of various complex systems by collecting sensor data, processing information, and executing operations. Intelligent systems typically include the following components.

- **Sensors and actuators:** Used for sensing and controlling the state and operation of physical systems.
- **Data collection and processing module:** Used to collect, process and store sensor data, extract features of the system, and make decisions based on those features.
- **Decision and control algorithms:** Using artificial intelligence technology to analyze and process the data and achieve intelligent control of the system by control algorithms.

Intelligent systems have numerous applications, including industrial automation, intelligent medical care, intelligent home, and intelligent transportation. The wide range of potential applications suggests that the use of intelligent systems will become more widespread in the future, driving innovation and progress in numerous industries.

### 3.1. Design and control of intelligent systems

The design process plays a crucial role in determining the performance, reliability, maintainability, and scalability of intelligent systems. In this section, we will provide an overview of the current research on intelligent system design and control.

**Figure 4:**
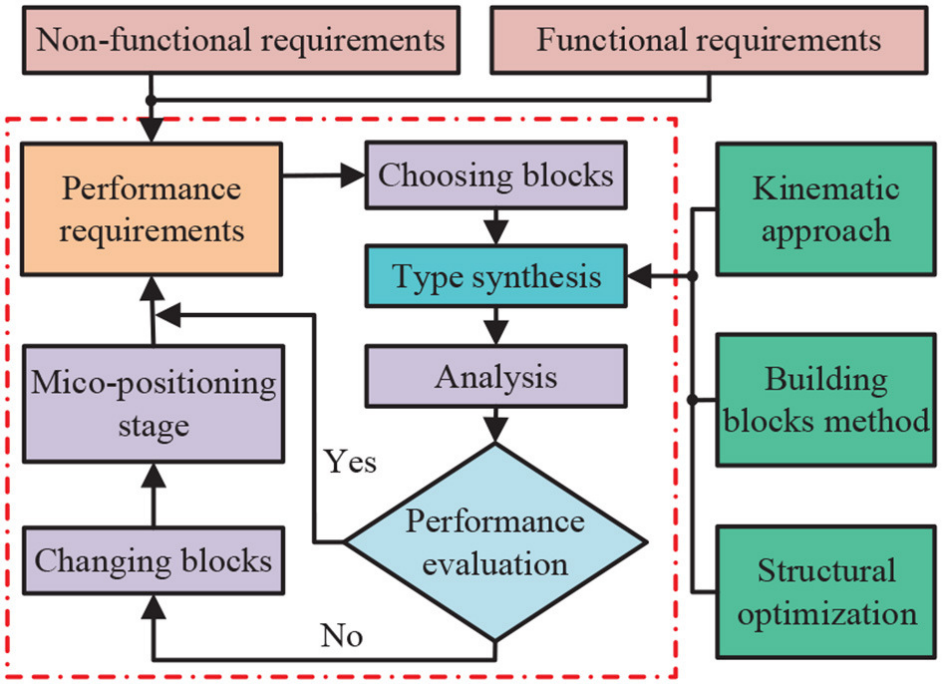
Detailed design framework of micro-positioning stages (MPSs), where the content in the red dotted box is the basic framework of MPSs.

In Ding et al. (2021), the authors proposed an intelligent system combining a pseudo-rigid body approach and a constant force output mechanism for workpiece contact force control. In this work, the intelligent system was constructed as a mathematical model and provided a theoretical analysis to verify it. To obtain the optimal parameters and structure, a particle swarm optimization (PSO) method was used and experimentally verified by the authors. As the paper concludes, this intelligent system is excellent and generalizable. In Lan et al. (2016), the authors studied an observer design method for fractional-order one-sided Lipschitz intelligent systems. Also, the asymptotic stability of the full-order observer error system has been ensured by using an indirect Lyapunov method and an equivalent model. In Ding et al. (2019a), the authors investigated a design scheme for a reconfigurable planar micro-positioning stages (MPSs) based on different functional modules, and details the flexibility and functionality of this scheme were presented in the paper. Finally, the authors point out that the system provides a new idea for the design of MPSs. Facing the practical need for higher precision MPSs (Liao et al., 2022e), the authors proposed a novel assembly concept (both planar and spatial configurations) that further improves the flexibility and functionality of intelligent systems. Figure 4 presents the detailed design framework of MPSs.

In Ding et al. (2022), an intelligent system of constant force mechanism based on the combination of negative and positive stiffness was presented. In this work, the authors have modeled and validated the system. The results of this paper indicate that in numerical experiments, this intelligent system can achieve the required constant force output and was consistent with the theoretical results. In addition, a class of semi-interactive intelligent systems has been proposed for the creation of robotic dance works (Peng et al., 2015, 2016). The authors point out that this system was capable of self-adaptive and self-learning capabilities and has been validated on the NAO robot with good performance. Besides the above instances, the intelligent system also has widespread application scenarios, such as equipment processing control (Tang et al., 2015; Wu et al., 2021), substation management (Hu et al., 2021), and UAV collaborative control (Xu et al., 2022).

### 3.2. Identification and detection in intelligent systems

Recognition and detection technology, integrated with computer vision technology and machine learning algorithms, has become a critical component of intelligent systems. The fundamental concept of this technology is to analyze, process, and comprehend input images or videos to identify and detect target objects or events. By accomplishing automatic recognition, classification, localization, and tracking functions, recognition and detection technology can augment the intelligence and automation of intelligent systems. It has extensive applications, including but not limited to, facial recognition, autonomous driving, and security monitoring. The development of recognition and detection technology relies on advancements in computer vision, machine learning, and signal processing techniques, which are enabling the creation of more efficient and accurate recognition and detection algorithms. Ongoing research is focused on enhancing the robustness, accuracy, and real-time performance of recognition and detection technology, thereby expanding its applicability to a diverse range of real-world scenarios (Qin et al., 2017; Hu et al., 2019b; Zhuo and Cao, 2021; Niu et al., 2022).

#### 3.2.1. Identification methods

In Zhuo and Cao (2022), the authors presented a novel approach for identifying damage in bolt connections of steel truss structures using sound signals. The proposed method employed support vector machine (SVM) classification, optimized with a genetic algorithm, to accurately recognize signals associated with bolt connection damage. The study demonstrated the effectiveness of SVM classification for signal recognition in structural health monitoring, specifically for detecting damage in bolt connections. In Wu et al. (2022c), a new scheme based on a low-strain pile integrity test and convolutional neural network (CNN) was proposed to identify concrete pile foundation defects with a remarkable accuracy of 94.4%. The authors described this method as more accurate, more reliable, and less destructive than traditional methods. Similarly, in Wu et al. (2022a), the authors proposed a method for the defect identification of foundation piles under layered soil conditions. In Tang et al. (2020), a human action recognition scheme was proposed, introducing and using the RGB-D image feature approach, which is a current research hotspot for effectively resisting the influence of external factors and improving the generalization ability of the classifier. The proposed scheme achieved excellent identification results on the public CAD60 and G3D datasets, utilizing three different patterns for human action feature extraction: The RGB modal information, based the histogram of oriented gradient (RGB-HOG), the depth modal information, based on the space-time interest points (D-STIP), and the skeleton modal information based on the joints' relative position feature (S-JRPF). In Xiang et al. (2018b), the authors identified Markov chains on trees (MCoT) through derivative constraints on the univariate distribution of sojourn time and/or hitting time, concluding that all MCoT can be identified using this method.

#### 3.2.2. Detection methods

In Luo et al. (2020), the authors investigated a novel chaotic system and its associated signal detection method, demonstrating high detection accuracy and noise immunity in experimental studies. The effectiveness and feasibility of the proposed method were verified through theoretical analysis, circuit simulation, and FPGA implementation, highlighting its potential as a reliable solution for signal detection in chaotic systems. In Wu et al. (2022b), a deep learning-based system was proposed for structural damage detection of engineering steel beams, where the vibration signals were used to extract features and detected by CNN. The experimental results show that the accuracy of this detection method achieved 95.14%. The authors concluded that this method has superior performance for structural damage detection of engineering steel beams compared to the SVM method. Furthermore, in Chen et al. (2022a), the authors provided a comprehensive review of the techniques for detecting code duplication in software development, analyzing the advantages and disadvantages of each approach.

### 3.3. Prediction and evaluation in intelligent systems

Prediction and evaluation are crucial elements in intelligent systems, facilitating accurate decision-making, pattern identification, model optimization, and goal attainment. These components interact with other aspects of intelligent systems, including learning algorithms and models, prediction and planning, evaluation and optimization, and self-adaptation and self-optimization, leading to enhanced system optimization and development.

#### 3.3.1. Prediction methods

Prediction is a crucial aspect of intelligent systems that can enable more informed decision-making, facilitate the discovery of regularities and patterns in data, optimize models, and support the attainment of system goals. Prediction can be achieved through the analysis of historical data to identify patterns and trends using intelligent systems. For instance, in Huang et al. (2022), the authors proposed a non-linear intelligent system for predicting the anti-slide pile top displacement (APTD) and identified multiple factors that affect the APTD. The proposed system was validated using four prediction methods, namely ELMAN, long short-term memory neural network (LSTM), support-vector regression (SVR), and maximal information coefficient-SVR (MIC-SVR), with results indicating superior performance in practical applications. Additionally, an integrated model based on wavelet transformation was introduced in Ding et al. (2013) for the prediction of both steady-state and dynamic-state network traffic. Low-frequency components were predicted using an improved gray theory, while the high-frequency components were predicted using a BP neural network algorithm, leading to increased prediction accuracy and reduced uncertainty. Moreover, an intelligent algorithm was introduced in Deng et al. (2019) for predicting the effective wind speed in wind turbines by considering the rotor speed, aerodynamic characteristics, and extreme learning machine. The authors reported that this algorithm is more efficient and accurate compared to traditional Kalman filter-based methods. Finally, an efficient search algorithm and optimization method were proposed in Song et al. (2020) to predict wind speed and extract the maximum wind energy.

#### 3.3.2. Evaluation methods

**Figure 5:**
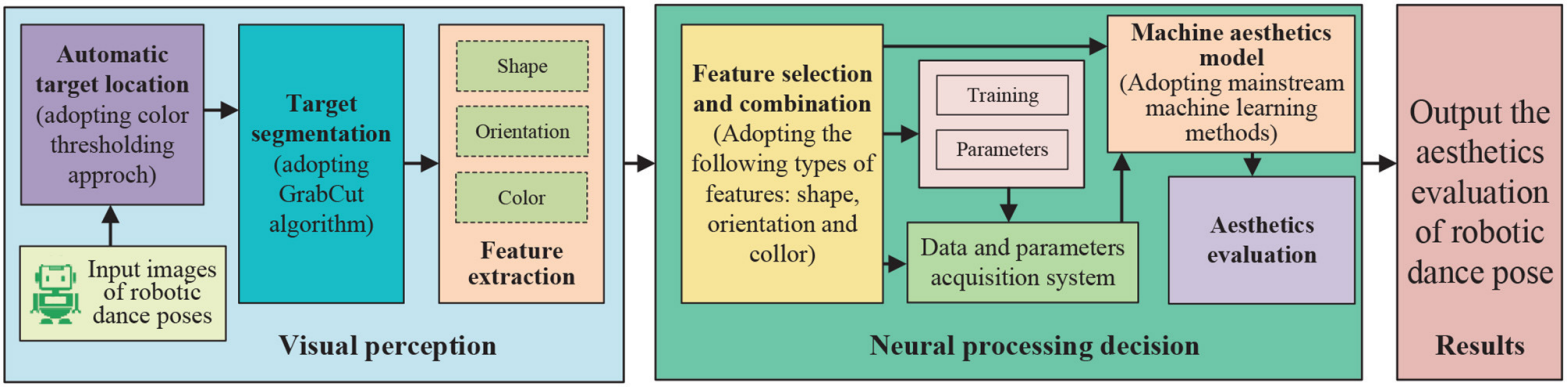
Computational framework for instantiating an aesthetic intelligence evaluation method for robotic dance poses. This evaluation method includes several components such as target localization, feature extraction, feature selection and combination, neural training, and decision-making.

Evaluation is a fundamental aspect of intelligent systems that allows for the assessment of the accuracy and performance of data, models, or decisions. During the evaluation process, the system compares actual values with ideal values to determine the accuracy and reliability of the model or decision. In the field of robotics, various methods have been proposed for the aesthetic evaluation of robotic dance movements. For instance, in Peng et al. (2022), the authors presented a method for aesthetic evaluation of robotic dance movements that employs key pose descriptors and integrated classifiers to train machine learning models. This method has been tested in a virtual environment and shown good performance. In Peng et al. (2019a), a brain-like intelligent system resembling the visual cognitive system of humans was proposed for the aesthetic evaluation of robotic dance poses. The system extracted features such as color, shape, and orientation and applied machine learning methods for evaluation. A computational framework for instantiating an intelligent evaluation method for robotic dance poses was presented in Figure 5. Similarly, in Li et al. (2020b), an automated method was proposed to evaluate the aesthetic level of robot dance movements by integrating multi-modal information. Features were extracted from visual and non-visual channels, and ten machine-learning algorithms were employed for evaluation, with the highest accuracy reaching 81.6%. Additionally, in Peng et al. (2019b), a feature fusion method was proposed for the automatic evaluation of robotic dance poses, which extracted four types of features, including color block, contour feature, region feature, and kinematic feature.

### 3.4. Intelligent communication systems

The intelligent communication system refers to a communication system that utilizes modern communication technology and artificial intelligence algorithms to dynamically adjust its parameters and structure based on varying communication needs, thereby achieving optimal communication performance and resource utilization efficiency. In this paper, we briefly describe three key aspects of the intelligent communication system: high-speed communication transmission methods, up-conversion mixer design, and spectrum sensing methods.

#### 3.4.1. High-speed communication transmission

In Sun et al. (2022), the authors proposed a method to enhance the rate range and reduce power consumption in high-speed serial links by utilizing an adaptive continuous time linear equalizer (CTLE) and a half-rate decision feedback equalizer (DFE) with a hybrid filter and a current-integrating summer. The system was tested using 10 Gb/s PRBS7 signals transmitted through an 18-inch FR4 backplane, and the post-simulation results demonstrated a rate range of 6.25-10 Gb/s with excellent performance. In Zhang and Yang (2020), the authors proposed an adaptive CTLE based on slope detection and a half-rate inferred DFE with intermediate frequency compensation and a small amount of equalization for the middle frequency range. The measurements showed an effective equalization loss of 24 dB at Nyquist frequency with a clear eye diagram at 36 Gb/s. Both works provide solutions to the challenges of high-speed transmission and offer valuable insights into the design of receiver equalizers for high-speed serial links.

#### 3.4.2. Up-conversion mixer design

In Chen et al. (2013), a folded up-conversion mixer was proposed by the authors, which employs a current reuse technique and achieves a conversion gain of 9.5 dB at a 1 V supply voltage while consuming only 258 μW of power. In Jin et al. (2014b), the authors presented a sub-harmonic up-conversion mixer that halves the required local oscillator frequency and achieves a higher conversion gain of 14.4 dB, albeit at the cost of increased power consumption of 1.65 mW at 1 V supply voltage. In Jin and Yu (2013), a current-reuse current-mirror-switch mixer was investigated by the authors, which features 8.5 dB conversion gain, 1.16 mW power consumption, lower supply voltage, higher linearity, and smaller chip area. All three works proposed novel mixers for wireless applications using 0.18-micron radio-frequency CMOS technology, with a focus on high performance, low power consumption, and small chip area, albeit with differences in specific technologies and performance metrics.

#### 3.4.3. Spectrum sensing

In Yang et al. (2017), the authors investigated a multi-band spectral sensing method based on eigenvalue ratios, which employs random matrix theory to determine the distribution of new statistics solely in the presence of noise. This approach allows for the reliable establishment of theoretical thresholds and exhibits superior performance in small sample scenarios. In Lei et al. (2016), the authors introduced a blind broadband spectrum sensing algorithm based on principal component analysis. This algorithm transforms the wide-band spectrum sensing problem into a sequential binary hypothesis test utilizing a generalized likelihood ratio test, enabling simultaneous operation on all sub-bands and overcoming noise uncertainty issues. Both studies propose innovative approaches to addressing the multi-band spectral perception challenge, without requiring prior knowledge. The authors emphasized the practical significance of these methods for applications such as radio spectrum allocation, spectrum sharing, and dynamic spectrum access.

### 3.5. Intelligent oscillator systems

Intelligent oscillation systems are complex devices designed to generate controlled vibration signals that exhibit adjustable amplitude and frequency. Generally, these systems comprise several essential components, including a vibration source (e.g., a motor or piezoelectric device), a controller, sensors, and feedback loops. With a diverse range of applications, these systems have demonstrated their effectiveness in areas such as structural vibration control, acoustic and mechanical system testing, and medical devices.

#### 3.5.1. Quadrature oscillator design

The quadrature oscillator is a passive oscillator that produces a sinusoidal wave with frequency and impedance determined by the inductor and capacitor values. This oscillator generates two orthogonal signals, sine and cosine waves, making it widely used in wireless communication systems. In Jin et al. (2015a), two variable frequency third-order quadrature oscillators (TOQOs) were proposed based on current differential transconductance amplifiers (CDTA). These TOQOs were completely resistorless and provided four quadrature current outputs at high output impedance terminals. In Jin and Liang (2013), a new resistorless current-mode quadrature oscillator based on CDTA was introduced, which provided two well-defined quadrature outputs at high-impedance terminals for easy cascading. Both works utilized CDTA for building the quadrature oscillator with the resistorless circuit, enabling monolithic integration, explicit orthogonal current outputs, direct cascading with other current-mode circuits, and controllable oscillation frequencies.

#### 3.5.2. Quadrature voltage-controlled oscillator design

The quadrature voltage-controlled oscillator (QVCO) is an active oscillator that generates a sinusoidal wave, where the oscillation frequency is determined by an external control voltage. QVCO typically consists of two orthogonal oscillation circuits, which can vary the oscillation frequency by altering the phase difference between the two circuits. In Jin and Tan (2019), the authors proposed a novel low-voltage and low-power QVCO that is coupled by four P&N transistors, yielding a wide tuning range and low phase noise while consuming a meager 2.31 mW. In Jin (2018a), the authors introduced a novel QVCO architecture that employs four capacitors to achieve enhanced phase noise and reduced power dissipation compared to conventional designs. Furthermore, Jin et al. (2014a) developed a programmable current-mode multi-phase voltage-controlled oscillator (MPVCO) using cascaded first-order all-pass filters, which provides multiple outputs. These studies have introduced significant advancements in the design of voltage-controlled oscillators, resulting in enhanced performance, compact size, and reduced power consumption. These advancements are crucial for numerous applications in wireless communication systems.

#### 3.5.3. Chaotic oscillator design

The chaotic oscillator is a non-linear dynamical system that exhibits complex, unpredictable behavior. It can be realized either through mathematical equations or physical circuits. In Jin (2018b), the authors proposed a novel digitally programmable multi-directional chaos oscillator (DPMDCO), which employs MOS switches for controlling the chaotic oscillation in three different directions. The DPMDCO achieves a compact size and low power consumption, making it suitable for practical applications. In Ouyang et al. (2022), a fully integrated chaotic oscillator (FICO) based on operational amplifiers and multipliers was presented. This system integrates all necessary circuit elements into a single chip, providing ease of implementation and compactness. Both DPMDCO and FICO were evaluated using the Cadence IC design tool, with DPMDCO consuming 99.5 mW at ± 2.5 V supply voltage and occupying 0.177 mm^2^ of chip area, while FICO consumed 148 mW and had a larger chip area of 6.15 mm^2^. These works demonstrate the potential for achieving compact and low-power chaotic oscillators through digital programmability and circuit integration.

### 3.6. Development directions and challenges

Intelligent systems are already being used in a wide range of applications, from virtual assistants and chatbots to self-driving cars and medical diagnoses. However, as these systems become more prevalent, they also face significant challenges, both in terms of technical limitations and ethical concerns. This section will explore the future of intelligent systems and the challenges they face.

#### 3.6.1. Development directions

Intelligent systems have been advancing at a rapid pace, and they will continue to transform our lives in the coming years. There are some of the most promising directions:

- **Healthcare:** Intelligent systems can help diagnose diseases, monitor patient health, and provide personalized treatment recommendations. In addition, intelligent systems can also be used to develop new drugs and therapies.
- **Transportation:** Self-driving cars are already being tested on public roads, and they have the potential to improve road safety and reduce traffic congestion. Intelligent systems can also be used to optimize transportation routes, improve logistics, and reduce carbon emissions.

#### 3.6.2. Challenges

Intelligent systems have the potential to transform our lives and revolutionize industries. However, they also face the following challenges:

- **Interpretability:** It is essential for intelligent systems to provide transparent and interpretable results, especially in critical decision-making processes. However, many of the state-of-the-art machine learning models are often considered “black-boxes,” making it difficult to understand how they arrived at their results. This lack of interpretability can hinder trust in the system.
- **Cybersecurity and privacy:** Intelligent systems collect, store, and process a vast amount of data, which makes them vulnerable to cyber attacks. There is also a risk of data breaches that may compromise the privacy and security of individuals.

## 4. Optimization algorithms and strategies

Optimization is a fundamental process of finding the optimal solution within a given set of constraints. In computer science, optimization algorithms constitute a class of algorithms employed to obtain the optimal solution, and they can be categorized into two types:

- **Stochastic algorithms:** The stochastic algorithms leverage random properties to achieve better solutions through corresponding probabilistic strategies. Such algorithms fall into the category of optimization algorithms in computer science. Examples of commonly used stochastic algorithms include genetic algorithms, particle swarm algorithms, and beetle antennae search algorithms (Khan et al., 2022a). While these algorithms can find near-optimal solutions in a relatively short time, they are not guaranteed to obtain the optimal solution.
- **Deterministic algorithms:** The deterministic algorithms always generate the same output for a given input. Linear programming, integer programming, and dynamic programming are some examples of deterministic algorithms. These algorithms can provide efficient solutions to optimization problems. However, their computational power and time may be limited when dealing with complex optimization problems.

Subsequently, we will present an overview of bio-inspired optimization algorithms and intelligent optimization strategies.

### 4.1. Bio-inspired optimization algorithms

Bio-inspired optimization algorithms are a type of stochastic algorithms that draw inspiration from the principles of biological evolution and swarm intelligence observed in nature. These algorithms aim to mimic the behavior of individual organisms or groups for solving complex optimization problems (Khan et al., 2020b, 2022c; Chen et al., 2022b).

#### 4.1.1. Particle swarm optimization (PSO) algorithm

**Figure 6:**
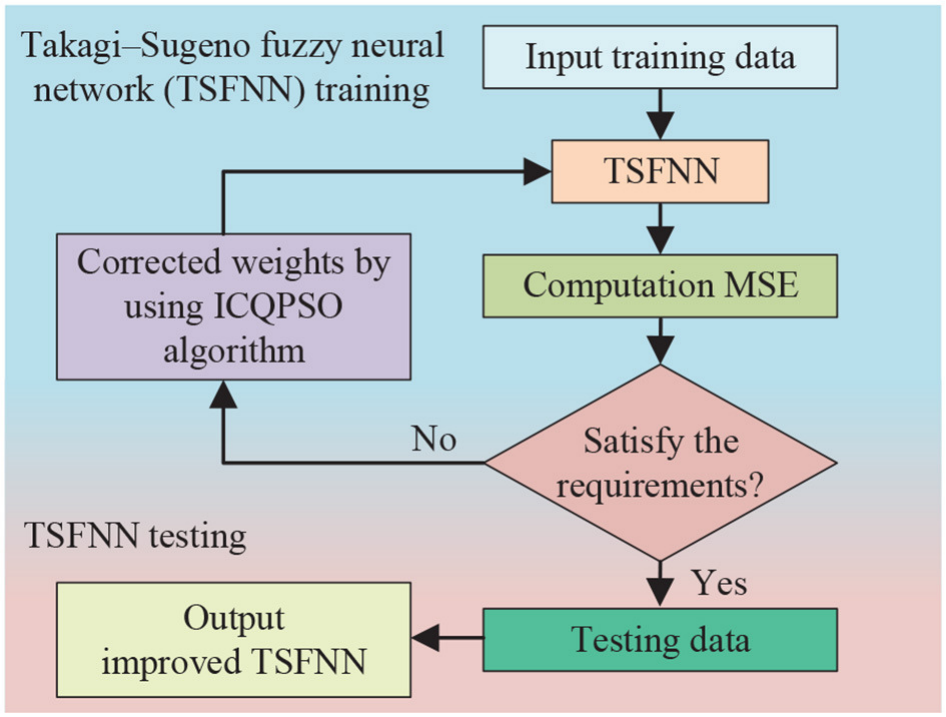
Training and testing flow chart for optimizing Takagi-Sugeno fuzzy neural networks (TSFNNs) by using an improved chaotic quantum particle swarm optimization (ICQPSO) algorithm. Mean square error (MSE) is a widely used metric to measure the average squared difference between the actual and predicted values of a regression problem. A lower MSE indicates that the predicted values are closer to the actual values, while a higher MSE indicates that the predictions are farther away from the actual values.

In a study by Peng et al. (2020), an enhanced chaotic quantum-inspired particle swarm optimization (ICQPSO) algorithm was introduced to address the issues associated with Takagi–Sugeno fuzzy neural networks (TSFNNs), such as slow convergence rate and extended computation time. The flow chart illustrating the training and testing process of the ICQPSO algorithm for optimizing TSFNNs can be found in Figure 6. In another study by Yang et al. (2022), an improved particle swarm optimization (IPSO) algorithm was proposed to identify the parameters of the Preisach model, which is utilized to model hysteresis phenomena. The authors demonstrated that the IPSO algorithm outperformed the traditional PSO algorithm in terms of faster convergence, reduced computation time, and improved accuracy.

#### 4.1.2. Genetic algorithm (GA)

In Ou et al. (2022), a hybrid knowledge extraction framework was developed by the authors, utilizing the combination of genetic algorithms and back propagation neural networks (BPNNs). An improved adaptive genetic algorithm (LAGA) was incorporated in the optimization of BPNNs. The efficacy of the LAGA-BPNNs approach was demonstrated through a case study involving the Wisconsin breast cancer dataset. Meanwhile, in Li et al. (2020a), the authors also investigated the applicability of the harmonic search algorithm to this knowledge extraction framework.

#### 4.1.3. Cuckoo search (CS) algorithm

In Zhang et al. (2021), the authors presented an improved cuckoo search (ICS) algorithm that addressed the limitations of the original cuckoo search (CS) algorithm. The proposed ICS algorithm incorporated non-linear inertial weight, which enhances the local optimization capability, and the differential evolution algorithm, which improves convergence accuracy. The performance of the ICS algorithm was evaluated, and it was found to outperform the original CS algorithm in terms of both global search and robustness. In Ye et al. (2022), the authors proposed an improved multi-objective cuckoo search (IMOCS) algorithm to solve multi-objective optimization problems. The IMOCS algorithm demonstrated good convergence performance by dynamically adjusting the balance between development and exploration, compared to existing CS algorithms. The proposed algorithm provides an effective approach to deal with multi-objective optimization problems, which often involve multiple competing objectives.

#### 4.1.4. Beetle antennae search (BAS) algorithm

**Figure 7:**
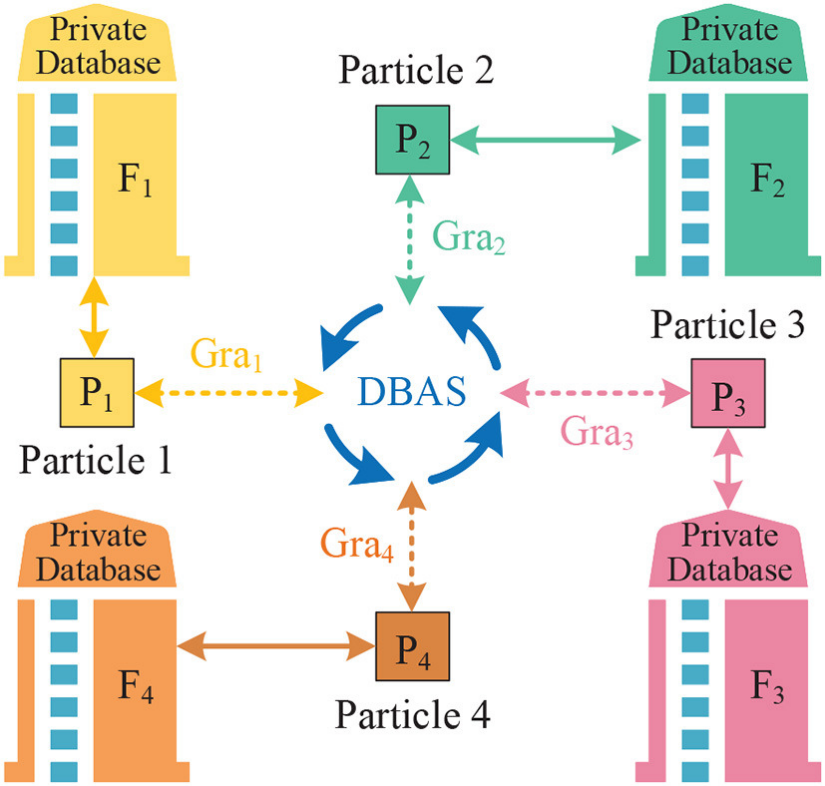
Framework of data exchange in the distributed beetle antenna search (DBAS) algorithm for solving multi-portfolio selection problem, where the search particles share only the gradient (Gra) and not the private information of the portfolio, such as customer information, stock information, and private databases.

In Khan et al. (2022d), a distributed beetle antennae search (DBAS) algorithm was proposed to solve the multi-portfolio selection problem, while ensuring privacy of investment portfolio data. The DBAS algorithm was shown to be efficient and robust in selecting the optimal investment portfolio. The paper also presented a data exchange framework for multi-portfolio selection, illustrated in Figure 7. In Liao et al. (2022c), the authors proposed a non-linearly activated beetle antenna search (NABAS) algorithm for fraud detection of publicly traded firms. They compared the performance of the NABAS algorithm to that of other popular methods, including the SVM-FK algorithm and the logistic regression model, and concluded that the proposed algorithm was more efficient and accurate for fraud detection. In Katsikis et al. (2021), a novel approach utilizing the BAS algorithm was proposed for solving the problem of time-varying mean-variance portfolio selection under transaction costs and cardinality constraints. This approach is based on state-of-the-art meta-heuristic optimization techniques and offers a more realistic solution to the problem as compared to conventional methods. The effectiveness of the proposed method was verified through numerical experiments and computer simulations, which demonstrated its superiority over traditional approaches. Overall, the study presents an online solution that addresses the limitations of static methods for solving time-varying financial problems.

### 4.2. Optimization strategies and systems

Optimization strategies and systems have become increasingly important across various fields as they offer effective solutions to complex problems by finding the best possible outcomes. In this subsection, we will provide an overview of the related research on optimization strategies and systems. Optimization strategies refer to the methods and techniques that are used to optimize a system or process. These strategies include but are not limited to heuristic algorithms, mathematical programming, and simulation-based optimization. Optimization systems, on the other hand, are computer programs or platforms that employ optimization strategies to solve complex problems. These systems can be standalone applications or integrated with other software tools. By exploring the latest research in optimization strategies and systems, we can gain a better understanding of how these techniques can be applied in different fields to improve efficiency, productivity, and overall performance.

#### 4.2.1. Optimization strategies

In Chen et al. (2014), the authors presented a cooperative obstacle avoidance model and an improved obstacle avoidance (OA) algorithm for mobile wireless sensor networks, aimed at enhancing the adaptability and robustness of the network in complex environments. The proposed strategies optimized path planning and achieved higher obstacle avoidance efficiency by predicting the motion path of obstacles and defining the steering direction. In Xiang et al. (2021), the authors proposed a new approach for automatic skeleton design that utilizes physical simulation and optimization algorithms to better adapt to various application scenarios. The paper concludes that the proposed optimization strategy outperforms other mainstream optimizers in robot design and animation applications.

#### 4.2.2. Optimization systems

The optimization system is a crucial tool to reduce the time and effort needed to find the optimal solution while guaranteeing its optimality. In Li and Zhang (2022), the authors presented an optimization system for generating benchmark dynamic test functions. The proposed system represents an advancement in the field of benchmark dynamic test functions, which is currently underdeveloped. In Deng et al. (2020), the authors proposed an optimal torque control system for controlling variable-speed wind turbines. As per the conclusion, this optimized system improved the effective wind speed estimation accuracy by 2%–7% and the efficiency of electrical energy generation by 0.35%. The proposed system offers a promising approach to enhancing the performance of wind turbines for electricity generation.

### 4.3. Development directions and challenges

Optimization algorithms and strategies have been widely used in various fields, including engineering, finance, and operations research, among others. The goal of optimization is to find the best solution to a problem within a given set of constraints. Optimization algorithms and strategies are continually evolving to meet the increasing demands of complex problems. This section will explore the future development and challenges of optimization algorithms and strategies.

#### 4.3.1. Development directions

Optimization algorithms and strategies are constantly evolving, driven by advances in mathematics, computer science, and various application domains. There are some potential directions that optimization algorithms and strategies may be headed:

- **Deep learning-based optimization:** Deep learning techniques such as neural networks have shown tremendous success in various applications, including optimization. One potential direction is to use deep learning techniques to optimize the parameters of optimization algorithms, making them more efficient and effective.
- **Optimization with uncertainty:** Many real-world optimization problems involve uncertainty, such as noisy measurements, incomplete information, or uncertain parameters. One potential direction is to develop new optimization algorithms that can handle uncertainty explicitly, such as robust optimization or stochastic optimization.

#### 4.3.2. Challenges

Despite the optimization algorithms and strategies have been widely developed and used, there are also significant challenges that need to be addressed:

- **Big data:** The growth of big data and the increasing complexity of data structures pose significant challenges for optimization algorithms and strategies. Dealing with large-scale, high-dimensional, and heterogeneous data requires advanced optimization techniques that can handle data efficiently and effectively.
- **Interdisciplinary applications:** Optimization problems are increasingly being used in interdisciplinary applications, such as healthcare, finance, energy, and transportation. These applications require optimization algorithms and strategies that can handle complex, multi-disciplinary problems, and that can effectively integrate domain knowledge, data analytics, and decision-making.

## 5. Conclusion

In this paper, we have analyzed and outlined the work related to neural networks, intelligent systems, and optimization algorithms and strategies in the rapidly evolving intelligence approach. Through an analysis and comparison of related work, we have shown that these intelligent approaches have rapidly evolved and have facilitated the efficient solution of practical problems. However, there are still emerging challenges that need to be addressed. Overall, this paper provides a valuable introduction and supplement to these important and rapidly evolving areas, highlighting their positive results and encouraging future research in these fields.

## Author contributions

BL and SL developed the initial idea for the paper. CH and BL conducted the literature review and analyzed the relevant studies. CH wrote the first draft of the paper. CH and XC reviewed and edited the paper. XC, BL, and SL provided supervision and support for the entire project and offering guidance and assistance throughout the writing process. BL secured funding for the paper. All authors have read and agreed to the published version of the manuscript.
